# A case of synchronous multiple bilateral breast cancer after breast augmentation

**DOI:** 10.1186/s40064-015-1615-1

**Published:** 2015-12-23

**Authors:** Shinya Yamamoto, Takashi Chishima, Fumi Harada, Yuka Matsubara

**Affiliations:** Department of Breast Surgery, Yokohama Rosai Hospital, 3211 Kodukue, Kouhoku-ku, Yokohama, 222-0036 Japan

**Keywords:** Breast cancer, Augmentation, Nipple-sparing mastectomy

## Abstract

Breast cancer after breast augmentation is not rare, but cases of bilateral breast cancer after augmentation are not often reported. A 43-year-old woman attended our hospital because of a mass in her left breast. She had undergone breast augmentation by implants 4 years before at a cosmetic surgery clinic. There were operative scars in her bilateral axilla. A detailed examination revealed bilateral breast cancer, and we performed nipple-sparing mastectomy in both breasts. Sentinel lymph node biopsy using dye was performed and it identified stained lymph nodes on both sides. The sentinel lymph node biopsy was negative for metastasis on both sides, so axillary lymph node dissection was not performed.

## Introduction

Breast cancer after breast augmentation is not rare, but cases of bilateral breast cancer after augmentation are not often reported. We present a rare case of synchronous multiple bilateral breast cancer after breast augmentation.

## Case report

A 43-year-old woman attended our hospital because of a mass in her left breast. She had undergone breast augmentation by implants 4 years before at a cosmetic surgery clinic. There were operative scars in her bilateral axilla.

Physical examination demonstrated a 15 mm hard mass located at the 10 o’clock position in the left breast. No palpable axillary lymph nodes were found, and the patient had no appreciable disease in the past. Her family history was negative for malignancy.

**Fig. 1:**
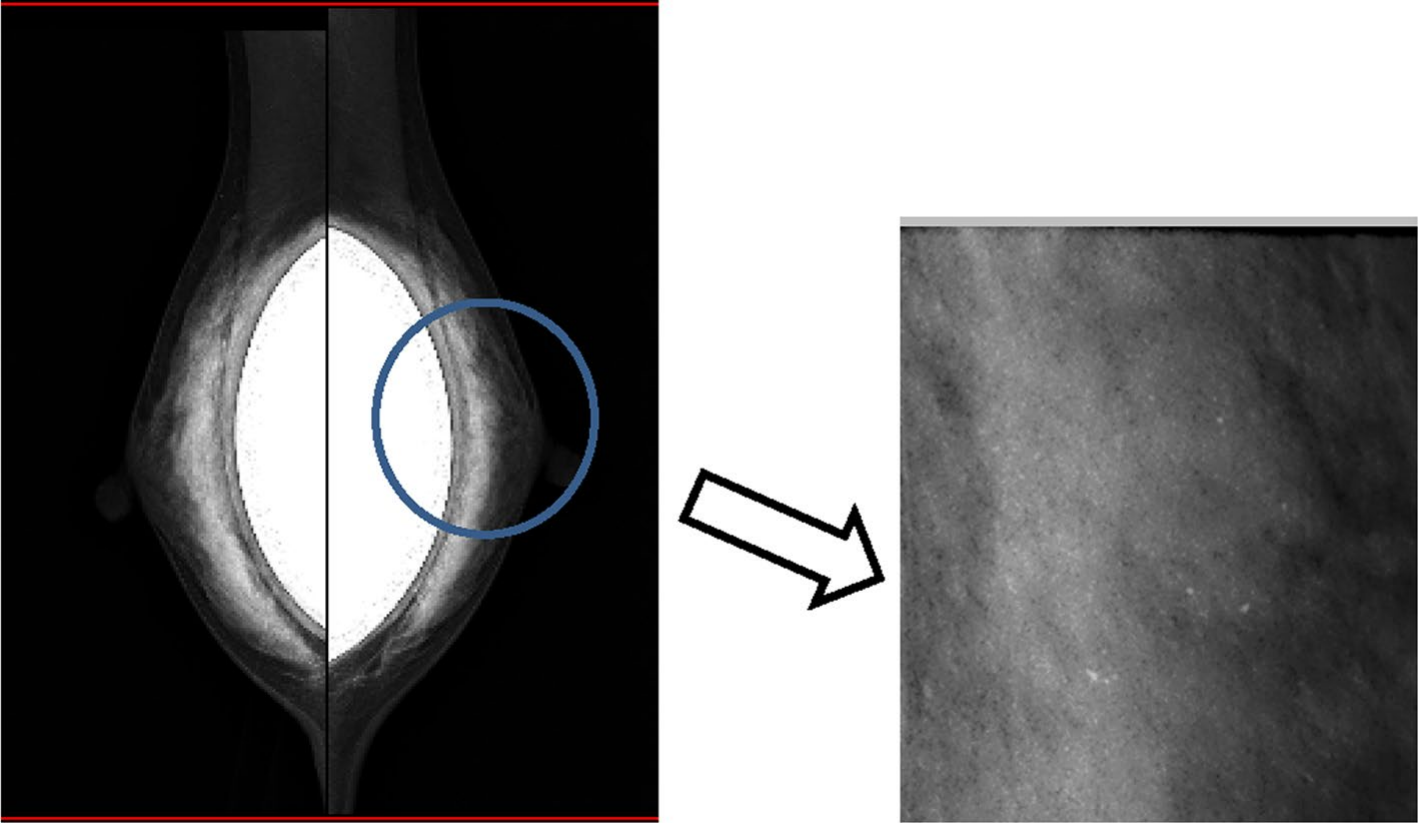
Mammography. Mammography of the left breast showed amorphous grouped calcifications in the left beast. There were no appreciable findings in the right breast

**Fig. 2:**
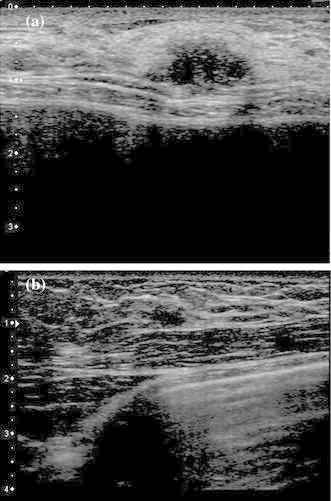
Ultrasound (US). US revealed a 15 mm hypoechoic mass with an irregular margin and rupture of the anterior borderline (**a**). US incidentally revealed a hypoechoic mass in the right breast (**b**)

**Fig. 3:**
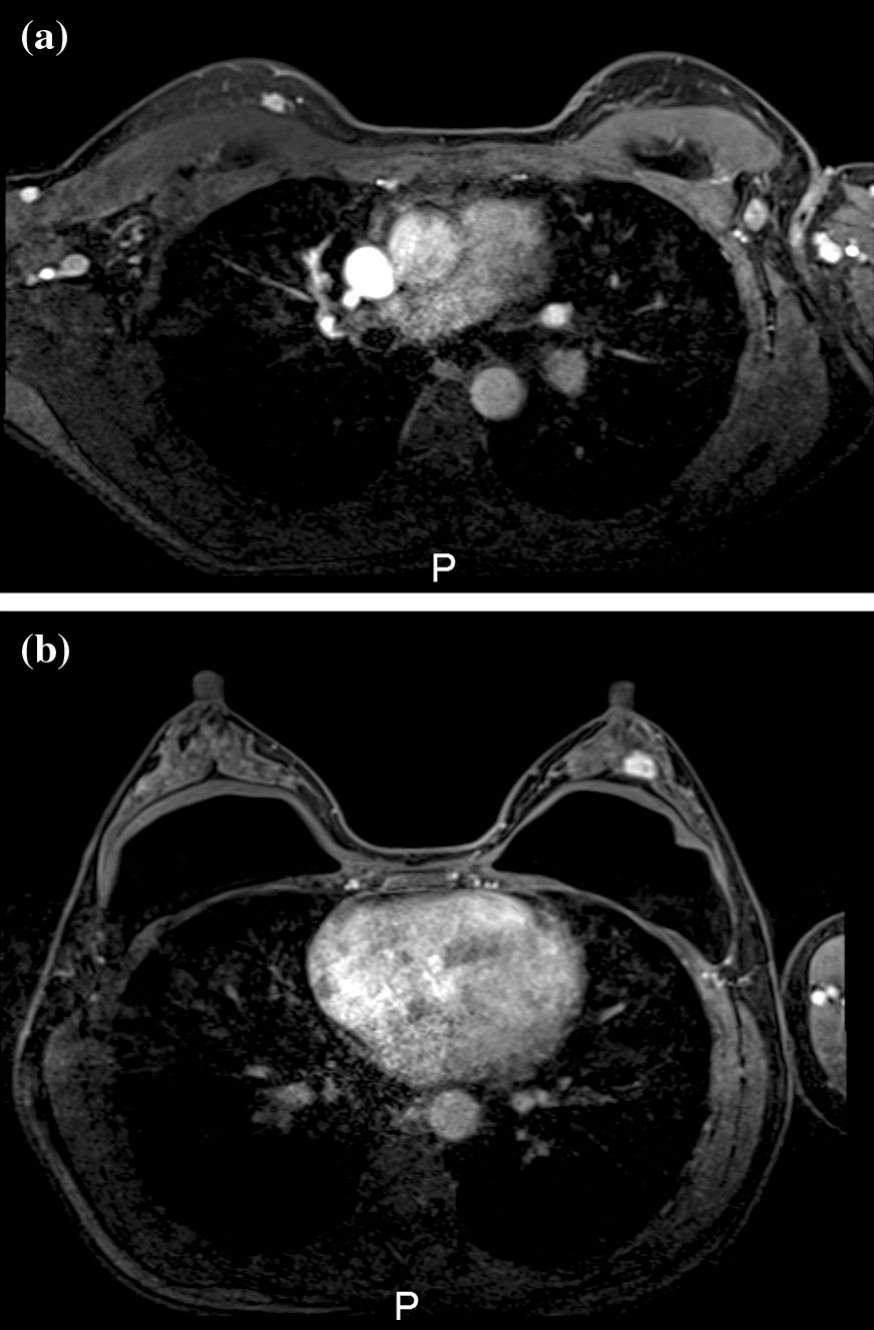
Magnetic resonance imaging (MRI). MRI showed enhancement of the lesion. There were implants under the pectoralis major muscle. **a** Right, **b** left

Mammography of the left breast showed amorphous grouped calcifications and there were no appreciable findings in the right breast (Fig. 1). Ultrasound (US) revealed a 15 mm hypoechoic mass with an irregular margin and invasion into the fat layer (Fig. 2a). US incidentally revealed a hypoechoic mass in the right breast (Fig. 2b). Magnetic resonance imaging (MRI), using a T1-weighted image, showed enhancement of the lesion (Fig. 3a, b).The left mass was located at the 10 o’clock position in the left breast, and the right mass was located at the 12 o’clock position in the right breast. The implants were located under the pectoralis major muscle.

Core needle biopsy was performed for bilateral masses and both masses were diagnosed as invasive ductal carcinoma.

It seemed as if a partial resection was performed because of the size of both tumors, but because the patient had already received breast augmentation and could not receive radiation therapy, we performed a nipple-sparing mastectomy (NSM) on both breasts. The entire mammary gland was removed in the layer where the pectoralis major muscle fascia was excised. It was removed without damaging the pectoralis major muscle and the capsule around the implants. If the implant capsule had been damaged, we planned to replace the patient’s implants with new ones, but there was no damage so the implants were preserved. The margin under the nipple was negative for metastasis on both sides. The weight of the specimen removed from the right side was 162 g, and that of the specimen removed from the left side was 148 g. Sentinel lymph node biopsy (SLNB) using dye was performed and it identified stained lymph nodes on both sides. The SLNB was negative for metastasis on both sides, so axillary lymph node dissection was not performed.

Pathological findings were as follows:

Right: The histological type was papillotubular carcinoma and the tumor size was 9 × 4 mm. There was another tumor on the lateral-caudal side. The histological type of this tumor was a scirrhous carcinoma and the tumor size was 4 × 2 mm. The first tumor was located at the 1 o’clock position and the nipple–tumor distance (NT) was 7 cm. A second tumor was located at the 12 o’clock position and NT was 5 cm. The tumors were separated by 2 cm. The immunostaining results for the first tumor were as follows: estrogen receptor (ER), 94 %; progesterone receptor (PgR), 96 %; HER-2, negative and Ki-67, 10 %.

Left: The histological type was scirrhous carcinoma and the tumor size was 13 × 7 mm. The immunostaining results were as follows: ER, 96 %; PgR, 97 %; HER-2, negative and Ki-67, 7 %.

Because the patient did not request breast cancer susceptibility gene (BRCA) testing, it was not performed. The patient is currently receiving hormone therapy, and she is free from recurrence.

## Discussion

Breast augmentation surgery is one of the most popular cosmetic procedures for women and breast cancer is the most frequent cancer among women (Stivala et al. 2012). Therefore, diagnoses of breast cancer in patients with breast augmentation will increase. However, in breast cancer after augmentation, there have been few reports of synchronous bilateral breast cancer; we found only one previous report (Johnson and Lloyd 1974).

There is no definitive consensus regarding the causal relationship between breast augmentation and breast cancer. However, several cohort studies have been analyzed and there is no breast cancer risk in women with prior augmentation (Handel 2007; Tuli et al. 2006).

A partial resection may have been performed because of the size of both tumors, but we choose NSM for several reasons. When radiation is performed with implants in place, the potential for implant capsular contracture is high; thus, the patient could not receive radiation therapy. In addition, because this patient had multiple tumors, lumpectomy was not recommended. We also determined that NSM was better than lumpectomy from the cosmetic aspect. Handel et al. reported that patients with augmented breasts who have with breast conservation treatment often experience poor cosmetic results and frequently require reoperation because of capsular contracture after radiation (Handel 2007).

The capsules that already existed in this patient were not damaged during NSM. Because the complication rate rises by breaking the capsules along with the replacement, we did not replace them.

It is important to note that the pectoralis major muscle and clavipectoral fascia is not damaged.

SLNB is the standard method for treating clinically node-negative breast cancer. However, the American Society of Clinical Oncology (ASCO) guidelines do not recommend SLNB in patients with prior nononcologic breast surgery because there is limited or insufficient data (Lyman et al. 2005). Conversely, Nagao et al. reported that SLNB was successfully performed in breast cancer patients with previous breast augmentation (Nagao et al. 2014). Rodriguez et al. also reported that past history of breast augmentation or reduction surgery is not a contraindication to the SLNB technique (Rodriguez Fernandez et al. 2009). They reported that the sentinel node identification rate was 100 % in the 70 patients in their study. In our patient, sentinel lymph nodes were identified on both sides using dye. SLNB results were negative for metastasis on both sides, so axillary lymph node dissection was not performed.

## Conclusions

We present a rare case of synchronous multiple bilateral breast cancer after breast augmentation.
